# Biocalorimetry for the biotechnological use of natural and synthetic macromolecules

**DOI:** 10.1007/s00253-026-13871-5

**Published:** 2026-05-21

**Authors:** Thomas Maskow, Hieu Linh Duong, Noelia Fernández Merayo, Dietmar Schlosser

**Affiliations:** 1 Department of Microbial Biotechnology, Helmholtz-Centre for Environmental Research - UFZ, Permoserstraβe 15, 04318 Leipzig, Germany; 2 Vietnamese-German University (VGU), Ring Road 4, Quarter 4, Thoi Hoa Ward, Ho Chi Minh City, Vietnam; 3 Department of Applied Microbial Ecology, Helmholtz-Centre for Environmental Research - UFZ, Permoserstraβe 15, 04318 Leipzig, Germany

**Keywords:** Biocalorimetry, Biothermodynamics, Lignocellulose, Plastics, Solid substrates, Synthetic polymers

## Abstract

**Abstract:**

Biocalorimetry offers a powerful approach for real-time process monitoring and optimization in the biotechnological utilization of both natural and synthetic macromolecules, particularly in complex solid-state systems aiming at the valorization of plant biomass or plastics’ waste. This review critically examines general strengths and limitations of biothermodynamics and calorimetry for monitoring microbial activity, which can be tracked across diverse scales and substrates via metabolic heat measurements. Metabolic heat-derived activity parameters enable the robust quantitative evaluation of the performance of microorganisms for substrate conversion, hence potentially representing valuable tools for bioprocess development and operation. While biocalorimetry is established in liquid-phase cultivation systems to some extent, its adaptation to solid-state fermentation, composting, and the biochemical breakdown of solid plastics still remains in early stages while holding promise for real-time control and upscaling. Challenges and limits of the applicability of this technology currently persist especially for mixed cultures and non-sterile processes. Nevertheless, expanding metabolic heat-based datasets to microbial functional traits could advance ecological and industrial applications. Overall, biocalorimetry is positioned as a valuable tool for advancing circular bioeconomy strategies, though further validation and methodological development are needed for broader adoption in both research and industrial contexts.

**Key points:**

• *Biocalorimetry reliably quantifies microbial activity on complex solid substrates*.

• *Biocalorimetry can support plant biomass and plastic waste valorization in a circular bioeconomy*.

• *Biocalorimetric monitoring is applicable from the laboratory to the technical scale*.

## Introduction

A substantial portion of the world’s organic carbon is found in polymeric form. Terrestrial plants alone contribute approximately 450 gigatons of carbon, predominantly in the form of polymers such as cellulose and lignin (Bar-On et al. 2018). These natural polymers represent a vast reservoir of carbon and energy. Furthermore, between 10 and 30% of the carbon content in fossil fuels is composed of polymers like kerogen and bitumen, highlighting their importance also in conventional energy production (Aslanov et al. 2022; Jiang et al. 2024).

The production of synthetic polymers continues to rise, with over 99% of plastics derived from fossil fuels. This trend raises serious environmental concerns, particularly regarding the degradation of macroplastics into harmful micro- or nanoplastics that infiltrate ecosystems (Alqahtani et al. 2023; Amobonye et al. 2021; Sangkham et al. 2022). Projections indicate that without substantial intervention, the mass of plastic in the oceans could surpass that of fish by 2050, emphasizing the urgent need to tackle plastic pollution (World Economic Forum 2016). Furthermore, microplastics have the capacity to adsorb harmful organic contaminants, thereby increasing their toxicity within marine food webs (Jambeck et al. 2014).

**Fig. 1:**
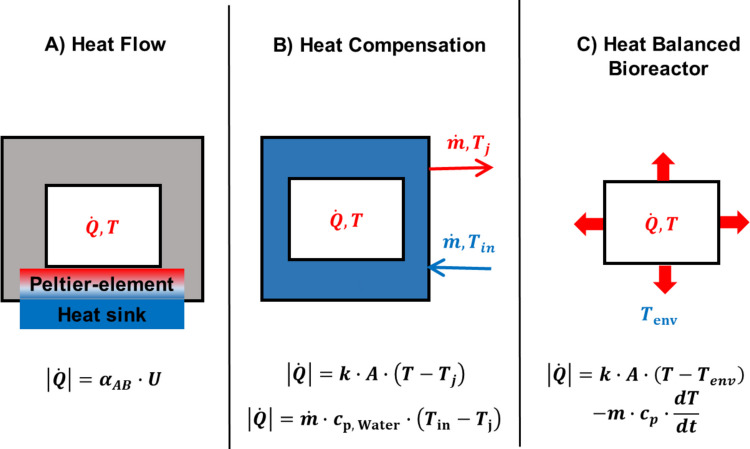
Principles of calorimetric process monitoring and control at different scales. The white region represents the heat producing culture, the grey region the insolation layer, and the blue region the heat sink. **A** Isothermal microcalorimetry (IMC), typically applied at laboratory scale, where heat production rate $\left|\dot{Q}\right|$ is interfered with the measured voltage *U*. **B** Reaction calorimetry at bench scale, where heat production rate is quantified from temperature differences, e.g., $\left(T-{T}_{\mathrm{j}}\right)$ or $\left({T}_{\mathrm{i}\mathrm{n}}-{T}_{\mathrm{j}}\right)$. *T*_j_ denotes the cooling jacket temperature, and *T*_in_ denotes the inlet temperature of the coolant. **C** Technical scale systems, where the heat production rate is derived from the reactor temperature *T* and the environmental temperature *T*_env_ typically via dynamic heat balances. The heat production rate $\left|\dot{Q}\right|$ is the target quantity in all cases. The corresponding proportionality factors are **A** the Seebeck coefficient ${\alpha}_{A\mathrm{B}}$, **B** either the product of heat transfer coefficient and exchange area ($k\cdot A)$ or the product of coolant mass flow rate and heat capacity ($\dot{m}\cdot {c}_{\mathrm{p}, \mathrm{W}\mathrm{a}\mathrm{t}\mathrm{e}\mathrm{r},}$, typically water as coolant), and **C** the effective heat capacity of the reactor contents ($m\cdot {c}_{p}$), relating $\left|\dot{Q}\right|$ to the temperature change rate (dT/dt), respectively. Conceptional representation adapted from calorimetric principles described in Altwasser et al. (2017) and Assael et al. 2022)

Conversely, synthetic polymers contain valuable carbon structures and binding energy that can be repurposed as raw material for new polymers or bulk chemicals, or utilized as an energy carrier within a circular economy. Biological processes involving bacteria, fungi, or enzymes show promise for harnessing these raw materials and promoting the sustainable use of synthetic polymers. However, microorganisms utilize a proportion of the carbon and energy content of the macromolecules for growth and maintenance of their metabolism, which limits the availability of these resources for other purposes. Similarly, organic substrates have to be invested in enzyme production, which altogether impacts the economic efficiency of biological processes aimed at macromolecule exploitation. In addition to efficiency considerations, the rate of biological reactions is also decisive. These reactions are significantly influenced by specific reaction conditions, e.g., temperature, buffer systems, water activity, or any solubility enhancers, which altogether aim to facilitate rapid conversion. Calorimetry emerges as a powerful tool for screening biocatalysts, optimizing process conditions, and enabling real-time monitoring of biological processes. If the energy content of a synthetic polymer is known based on, e.g., combustion calorimetry, process calorimetry can directly quantify its energy use efficiency since it measures the material’s metabolic or thermal energy conversion (Kästner et al. 2024) at different scales. Figure 1 depicts a generalized calorimetric process monitoring and control scheme ranging from laboratory (Fig. 1A: isothermal microcalorimetry (IMC)) via bench (Fig. 1B: reaction calorimetry) to finally technical scale (Fig. 1C: dynamically heat-balanced bioreactors). Dynamic enthalpy balances based on calorimetric monitoring allow to quantify the completeness of polymer hydrolysis (Vogel et al. 2021), assess the proportion of carbon and energy directed into new biomass, and measure reaction rates (Maskow and Paufler 2015). Furthermore, a thermodynamic state variable such as the measured enthalpy enables predictions grounded in thermodynamic principles (Assael et al. 2022; Kleerebezem and Van Loosdrecht 2010; von Stockar 2010). While emerging research is now beginning to explore these potentials for natural macromolecules (Cabaneros Lopez et al. 2019; Duong et al. 2024; Islam et al. 2022), potential applications towards synthetic macromolecules like plastics remain largely disregarded so far.

Despite the aforementioned benefits, calorimetry has been underutilized in the biotechnological exploitation of natural and synthetic macromolecules. This limitation may stem from the challenge associated with inhomogeneous systems and the use of solid or particulate substrates, which can complicate signal interpretation. To address this gap, this mini-review specifically focuses on applications involving solids. Other fascinating aspects of calorimetric techniques such as differential scanning (DSC), bomb or combustion, and indirect calorimetry, and their applications in other fields are not considered. A considerable range of alternative methods based on optical principles (e.g., scattered light, infrared, and Raman spectroscopy), electrochemical techniques (e.g., impedance and capacitance spectroscopy), or mass spectrometry-aided analyses of volatile or soluble (bio)chemicals, which also offer the possibility of non-invasive measurements and/or the provision of bioprocess data in real time, exists beyond the scope of the overview presented here (for recent reviews please refer to, e.g., Dambruin et al. 2025, Nikita et al. 2023 and Zhang et al. 2019). Consequently, this mini review aims to (i) analyze the current applications of biothermodynamics and calorimetry in the valorization of natural macromolecules, (ii) derive potential applications for synthetic macromolecules based on this analysis and compare them with pre-existing pioneering work, (iii) discuss related challenges and limitations, and (iv) identify future research needs. 

## Advantages of calorimetry

Calorimetry offers distinct advantages, making it an invaluable tool across diverse fields of research and applications. One key benefit is its ability to perform non-invasive real-time measurements with exceptional sensitivity. Continuous measurements often outperform conventional methods based on discontinuous sampling by providing superior parameter optimization, in particular for non-linear, exponential growth functions. This advantage is especially pronounced in the fast screening of environmental influences, dose dependencies, and similar variables (Braissant et al. 2010b). Sensitivity is crucial for the quantification of activities of only a few microbes or slow-growing microbial communities and the detection of subtle metabolic changes. In such cases, nanocalorimetry, which can detect power levels as low as a few nanowatts, proved highly effective (Robador et al. 2018, 2016).

Another strength of calorimetry is its independence from scale (Maskow and Paufler 2015). This technology is efficiently functioning across a wide volume range from the nanoliter to the cubic meter scale, as demonstrated in various biotechnological and industrial contexts. Innovations like chip calorimeters integrate all essential calorimetric functions onto compact electronic platforms allowing the measurements of the metabolism of just a few cells (Lerchner et al. 2017; Vehusheia et al. 2023a). The ratio of heat producing volume to heat exchanging surface increases upon upscaling the heat producing volume. This increasing ratio facilitates efficient bioprocess control even at large technical scales (Kottelat et al. 2021; Schuler et al. 2012; Türker 2003).

To estimate the effect of scaling, a well-mixed cylindrical bioreactor may be considered. The volume-specific metabolic heat production rate, ${\dot{Q}}_{V}=\dot{Q}/V$ (in W/L), contributes to both temperature rise, dT/dt (in K/s), and heat exchange with the environment ($k\cdot A\cdot \left(T-{T}_{\mathrm{e}\mathrm{n}\mathrm{v}}\right)$). Assuming that the heat capacity of the reactor itself is small in comparison to the reactor content, the energy balance can be written as follows:

1 $$\rho \cdot {c}_{\mathrm{p}}\cdot V\cdot \frac{\mathrm{d}T}{\mathrm{d}t}={\dot{Q}}_{V}\cdot V-k\cdot A\cdot \left(T-{T}_{\mathrm{e}\mathrm{n}\mathrm{v}}\right)$$

where $\rho$ and ${c}_{\mathrm{p}}$ are the density and heat capacity of the reactor content, respectively, and *V, k, A*, and ${T}_{\mathrm{e}\mathrm{n}\mathrm{v}}$ are the volume of the reactor content, heat transfer coefficient, heat exchanging area, and environmental temperature, respectively. Rearranging Eq. 1 highlights the influence of the scaling through the A/V ratio):

2 $$\frac{\mathrm{d}T}{\mathrm{d}t}=\frac{{\dot{Q}}_{V}}{\rho \cdot {c}_{\mathrm{p}}}-\frac{k}{\rho \cdot {c}_{\mathrm{p}}\cdot }\cdot \frac{A}{V}\cdot\left(T-{T}_{\mathrm{e}\mathrm{n}\mathrm{v}}\right)$$

The first term in Eq. 2 is independent from the heat exchange with the environment while the second term directly depends on the A/V ratio. For a cylindrical bioreactor, A/V equals the sum of 2/r and 2/h. As the reactor is scaled up (increasing radius *r* or height *h*), the contribution of the second term to the temperature rise decreases. This means that larger bioreactors operate closer to adiabatically, enabling more accurate calorimetric monitoring.

The versatility of calorimetry becomes also obvious from its application for the study of an extensive array of biological systems, ranging from biomolecular interactions and enzyme activities to bacteriophages (Morais et al. 2013; Xu et al. 2018a), bacteria (Gysin et al. 2018), microbial communities, fungi, plants (Burnett and Grobe 2013), and even large animals (Kaiyala and Ramsay 2011; Walsberg and Hoffman 2005). This flexibility makes calorimetry particularly well suited for exploring complex ecological and physiological systems.

Moreover, calorimetry can be applied to different matrices. It can monitor microbial activities in liquid cultures, on or in agar plates (Braissant et al. 2010a; Fricke et al. 2019), of biofilms (Astasov-Frauenhoffer et al. 2014; Buchholz et al. 2010), on filters (Brueckner et al. 2017; Solokhina et al. 2017), on wood (Wadsö et al. 2017b), on titanium disks (Astasov-Frauenhoffer et al. 2014), and even in soil systems (Barros et al. 2023, 2007), hereby providing valuable insights into the energy dynamics and microbial interactions within these diverse environments.

A unique advantage of calorimetry lies in its direct measurement of energy flows. Unlike other methods that require complex calculations to estimate energy transformations in heterogeneous or compositionally complex substrates, calorimetry captures these flows directly and accurately. The calorimetrically determined reaction heats can quantitatively be linked to yield coefficients for biomass or potential products via the law of Hess. Although this approach can be applied to a multitude of products as, e.g., demonstrated for compatible solutes (Schumer et al. 2007), its application becomes more challenging in more complex systems such as soil (Kästner et al. 2024).

Additionally, calorimetry provides access to fundamental thermodynamic state functions such as enthalpy, Gibbs energy, and entropy (Assael et al. 2022). The resulting insights enable predictive modeling, which has proven effective in applications ranging from biotechnological optimization of cell factories to ecological studies of energy constraints in microbial and environmental systems (Cossetto et al. 2025; Heijnen and Kleerebezem 2010; Maskow and von Stockar 2005; Smeaton and Van Cappellen 2018). Table 1 provides examples for successful applications of calorimetry.

**Table 1:** Examples of (bio)calorimetry applications for various purposes

Field of application	Biological test system	Aims	Calorimeter type, manufacturer, city, country	MNC^a^	V_R_^b^ (mL)	LOD_V_^c^ (mW/L)	Main findings	References
Dentistry	*Streptococcus sanguinis*	To investigate dental plaque-initiating bacterial adhesion to human tooth surfaces and dental materials	TAM III, TA Instruments, New Castle, DE, USA	24	4	0.05	Microcalorimetry offers a rapid, reproducible approach to study bacterial adhesion to dental materials	Hauser-Gerspach et al. (2008)
Agriculture	Seeds and soil microbiome	To monitor toxic effects of ionic liquids on seed germination and soil microbial communities	TAM III, TA Instruments, New Castle, DE, USA	24	4	0.05	Choline dihydrogen phosphate shows diverse but overall non-toxic effects on seed germination, soil microbes, and soil respiration	Cruz et al. (2024)
Medicine	Cancer cells	To monitor metabolic activities of cancer cells	calScreener™, SYMCEL, Stockholm, Sweden	36	0.6	0.2	Isothermal calorimetry shows promise to transform cancer research, diagnosis, and treatment	Bayode et al. (2023)
Soil science	Microbial communities	To quantify microbial carbon and energy use efficiency of substrate addition to arable soils	TAM Air, TA Instruments, New Castle, DE, USA	8	20	0.2	A mass and energy balance of cellulose degradation in arable soils could be provided	Wirsching et al. (2025)
Sanitation	*Legionella pneumophila* in water samples	To determine bacterial water contamination with *Legionella pneumophila*	TAM III, TA Instruments, New Castle, DE, USA	12	4	0.05	Early, reliable detection of water contamination with *Legionella pneumophila* contamination	Fricke et al. (2020)
Biosafety assessment	*Escherichia coli* containing lambda prophages	To study activation of silent viruses in bacteria by different chemicals	TAM III, TA Instruments, New Castle, DE, USA	12	4	0.05	Activation of silent bacteriophages can be quantified by calorimetry	Xu et al. (2018a,b)
Biotechnology	*Blakeslea trispora*	Monitoring and optimization of solid-state fermentation processes	Mc-Cal/100P, C3 Prozess- und Analysentechnik, Haar, Germany	12	20	1	Simple, cost-efficient monitoring of growth, product formation, and overheating risks possible	Altwasser et al. (2017)
Lab-on-chip technologies	*Escherichia coli*	To screen cell populations	Chip calorimeter, Micro and Nanosystems, Department of Mechanical and Process Engineering, ETH Zurich, Zurich, Switzerland	2	0.046	1.7	Efficient microbial activity measurement at the microscale, enabling integration with lab-on-chip systems	Vehusheia et al. (2023b)
Electrobiotechnology	Electroactive microorga-nisms	To discover unknown energy sinks in electrobiotechnology	CPA 202 HighSens Reactor, CemiSens, Microdidakt AB, Malmö, Sweden	1	250	0.4	Heat quantification at biofilm electrodes, revealing a microbial electrochemical Peltier effect as a significant energy loss in microbial electrochemical systems	Korth et al. (2016)
Photobiotechnology	Microalgae	To quantify energy efficiency of photosynthesis	2247 Thermal Activity Monitor, Thermometric AB, Järfälla, Sweden	4	20	0.05	Photosynthetic efficiency quantified via heat and oxygen flux measurements in real time	Linke et al. (2026)

^a^*MNC*, maximum number of channels

^b^*V*_*R*_, volume of the reaction vessel

^c^*LOD*_*V*_, minimum volume-related thermal limit of detection

All together, these combined advantages highlight the role of calorimetry and thermodynamics as a powerful tool for advancing process understanding in biotechnology, systems biology, ecology, and beyond.

### Biocalorimetry towards bioprocess development and operation

#### Usefulness of metabolic heat-derived process parameters

Generally, biocalorimetric data need to be adequately translated into usable biological data to enable their interpretation (Braissant et al. 2010b, 2013; Wadsö 1986). Metabolic heat production rates derived from biocalorimetric measurements of metabolically active microbial cells can be used to estimate microbial growth rates. A prerequisite for this is a sufficiently close correlation between the heat production rate and viable cell counts. Such a correlation is typically observed during exponential growth, where the specific heat production rate per cell represents a proportionality factor linking both types of data. Typical values of cell-specific heat production rates are, e.g., 0.05 pW cell^−1^ for resting *E. coli* cells, 0.2 pW cell^−1^ for anaerobically growing *E. coli*, 0.8 pW cell^−1^ for aerobically growing *E.* coli, or 40 pW cell^−1^ for the fungus *Fusarium roseum* (Maskow et al. 2011). For the determination of microbial growth rates on the basis of calorimetric measurements, the specific heat production rate per cell needs to be known and remain sufficiently constant over the considered period of time (Braissant et al. 2013).

Metabolic heat production rates can be integrated over time to yield growth reaction enthalpies. Under appropriate boundary conditions, these reaction enthalpies can be converted into biological equivalents such as biomass or metabolic products (Braissant et al. 2010b, 2013; Wadsö 1986). To derive microbial growth curves and thereby also growth rates, lag phases, and other growth characteristics, constant cell-specific proportionality factors are required to translate heat data signals into biomass. Here, one approach is to use the heat content of cellular biomass, which is typically expressed in kJ/mol of a “unit carbon formula” (UCF). For constructing a valid UCF, cells must be grown exponentially and be free of storage compounds (Battley 2003; Braissant et al. 2013; Zwietering et al. 1990). Typical UCF examples are C_1_H_1.613_O_0.557_N_0.158_P_0.012_S_0.003_K_0.002_Mg_0.003_Ca_0.001_ for *Saccharomyces cerevisiae* (Battley 1999) and C_1_H_1.571_O_0.429_N_0.143_ for microbial soil communities (Kästner et al. 2024), among other figures published for several microbial strains by, e.g., Popovic (2019).

**Fig. 2:**
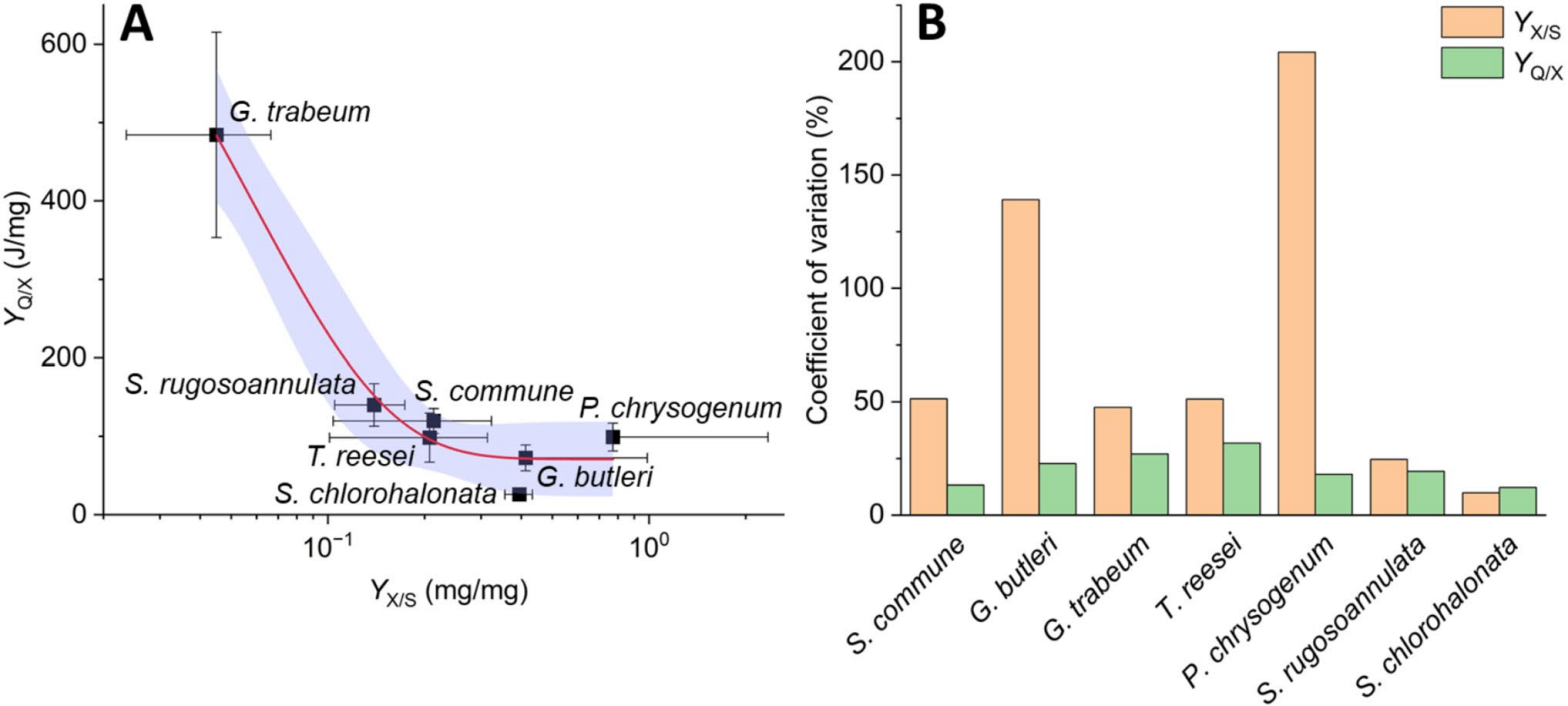
Metabolic heat yield coefficients (*Y*_Q/X_) vs. biomass yield coefficients (*Y*_X/S_) of seven filamentous fungi during aerobic growth at 28 °C on 0.5 g milled wheat straw (about 2 mm particle size; initial moisture content 500%, on a dry mass basis) in 40-mL calorimetric propylene vials placed in a MC CAL® isothermal microcalorimeter (C3 Prozess- und Analysentechnik GmbH, Haar b. München, Germany) (**A**), and corresponding coefficients of variation (%) observed for the *Y*_Q/X_ and *Y*_X/S_ values, respectively (**B**). The investigated fungal species are assigned to the corresponding data points (black squares) in (**A**) and to corresponding coefficients of variation in (**B**). Data points and error bars in (**A**) represent means and standard deviations for triplicate fungal cultures, respectively. Coefficients of variation in (**B**) were calculated as the standard deviation divided by the mean, multiplied with 100. The solid red line in (**A**) results from exponential fitting employing the software OriginPro 2024b (OriginLab Corp., Northampton, MA, USA) (coefficient of determination *R*^2^ > 0.97) Logarithmic scaling of the x axis in (**A**) was chosen to facilitate reading. All experimental data were taken from Duong et al. (2022a, b)

Metabolic heat yield coefficients (*Y*_Q/X_) representing the cumulative metabolic heat released (*Q*) per biomass unit (*X*; expressed as dry mass), which previously have been determined, e.g., during growth of different filamentous fungi on a solid lignocellulosic substrate such as wheat straw, can also be used to aid the interpretation of metabolic heat data (Duong et al. 2022a, b). *Y*_Q/X_ values are species-specific and allowed to, e.g., model fungal growth on very heterogeneous solid substrates and to deduce various growth-related fungal activity parameters (Birou et al. 1987b; Duong et al. 2022a, b; Mohan and Sivaprakasam 2017; Schuler et al. 2012; Sivaprakasam et al. 2011). Even more, they also seem to link fungal potentials for biomass production to the corresponding ecological strategies employed during fungal resource utilization (Duong et al. 2022a, b). This last point comes from the fact that *Y*_Q/X_ values inherently indicate the relationship between resource channeling into biomass production and other functional attributes, e.g., exoenzyme or metabolite production (Duong et al. 2022a, b). Qualitatively, comparable information can also be retrieved from biomass yield coefficients (*Y*_X/S_) based on biomass yields (*X*) and consumed substrates (*S*). Pairs of *Y*_Q/X_ and *Y*_X/S_ values previously experimentally determined for various filamentous fungi are exemplified in Fig. 2.

Remarkably, based on the comparison of several species-specific fungal *Y*_Q/X_ and *Y*_X/S_ values derived from fungal growth on solid lignocellulose, *Y*_Q/X_ data seem to be more reliable than their respective *Y*_X/S_ counterparts because of their apparently higher robustness and less susceptibility to errors (Fig. 2A and B) (Duong et al. 2022a, b). Also, a more meaningful interpretation of *Y*_Q/X_ values compared to *Y*_X/S_ data is suggested since *Y*_X/S_ values are usually calculated based on the disappearance of a presumable growth substrate in the understanding that the substrate disappearance is solely caused by the biocatalytic activity of the organism of concern, hereby frequently ignoring other potential causes of substrate disappearance, e.g., physico-chemical ones. Instead, metabolic heat production rate measurements provide a more direct proof for biochemical substrate conversion (Duong et al. 2022a, b).

It should nevertheless be noted that the heat production rate represents a nonspecific sum parameter potentially integrating signals of all (bio)chemical and (bio)physical processes simultaneously going on in the system under consideration, which may cause considerable limitations to the information value of related data (Braissant et al. 2010b; Wadsö 2002). However, calorimetric records can become more informative if combined with results of accompanying specific analyses on the same reaction system (Wadsö 2002) as illustrated in more detail for growth-coupled product formation below. Also, sufficiently well-understood biological phenomena can be monitored with high accuracy despite the potential drawback of nonspecificity (Braissant et al. 2010b). For instance, distinct heat flow patterns displaying more than one maximum over time can indicate metabolic shifts, such as the successive utilization of different carbon sources resulting in diauxic growth, a transition from aerobic respiration to anoxic fermentation or even a mixed respiro-fermentative metabolism (Braissant et al. 2010b, 2013; Maskow et al. 2014). Also, the growth of mixed populations may become visible in this way (Braissant et al. 2013). Preferences and tolerances of (micro)organisms with regards to nutrient requirements and further environmental or cultivation conditions and their sensitivity towards potentially toxic or inhibiting compounds can, e.g., be examined via determination of the duration of lag phases and growth rates with the help of appropriate growth models, which are applicable to calorimetric data (Braissant et al. 2013).

To differentiate between growth and parallel product formation, the heat production rate from product-forming strains can, in the simplest case, be compared with that from non-product-forming strains (Regestein et al. 2013). Combining heat measurements with respirometric data is another possible approach that is easy to implement in a calorimetrically monitored bioreactor. Recently, a conventional isothermal microcalorimeter (IMC) was equipped either with a CO_2_ monitoring sensor (Yang et al. 2026) or an established respirometer with calorimetric functions (Fricke et al. 2024, Di Lodovico et al. 2026). To exemplify the additional information that could be derived by such approaches, the ratio of heat production rate to CO₂ evolution rate changes from 200 kJ/mol CO_2_ to 48.1 ± 3.6 kJ/mol upon the onset of alcoholic fermentation during the growth of *S. cerevisiae* (Fricke et al. 2024).

If more information is available for, e.g., reaction calorimeters or calorimetrically monitored bioreactors, substrate consumption-related balances of microbial processes involving—among other parameters—product yields can be established as follows (Pirt 1982):

3 $${r}_{\mathrm{S}}= \frac{\mu \cdot {C}_{\mathrm{x}}}{{Y}_{X/S}^{Max}}+{m}_{\mathrm{E}}\cdot {C}_{\mathrm{X}}+\frac{{r}_{\mathrm{P}}}{{Y}_{P/S}^{Max}}$$

Here, *r*_s_ equals the substrate consumption rate, *µ* is the specific growth rate, *m*_E_ denotes the substrate-related maintenance coefficient, *C*_x_ represents the biomass concentration, *r*_p_ is the product formation rate, and ${Y}_{X/S}^{Max}$ and ${Y}_{P/S}^{Max}$ denote the maximum yield coefficient for biomass and product formation, respectively. If both sides of Eq. 1 are divided by the growth rate ($\mu \cdot {C}_{\mathrm{x}})$, a relationship that only contains the yield coefficients is obtained (Eq. 4):

4 $$\frac{1}{{Y}_{\mathrm{X}/\mathrm{S}}}=\frac{1}{{Y}_{X/S}^{Max}}+\frac{{m}_{\mathrm{E}}}{\mu }+\frac{{Y}_{\mathrm{P}/\mathrm{X}}}{{Y}_{P/S}^{Max}}$$

If it is possible to define the substrate and the product, a simple mathematical link between *Y*_Q/X_ and *Y*_X/S_ can be written as follows (Birou et al. 1987b):

5 $${Y}_{\mathrm{Q}/\mathrm{X}}=\frac{{\Delta}_{\mathrm{C}}{H}_{\mathrm{S}}-{Y}_{\mathrm{P}/\mathrm{S}}\cdot {\Delta}_{\mathrm{C}}{H}_{\mathrm{P}}}{{Y}_{\mathrm{X}/\mathrm{S}}}-{\Delta}_{\mathrm{C}}{H}_{\mathrm{X}}+{X}_{3}\cdot {\Delta}_{\mathrm{C}}{H}_{\mathrm{N}}$$

with *∆*_C_*H*_S_*, ∆*_C_*H*_P_*, ∆*_C_*H*_X_,and *∆*_C_*H*_N_ representing the combustion enthalpies of substrate, product, biomass, and the nitrogen source, respectively. *X*_3_ denotes the number of nitrogen atoms according to the UCF of biomass. Equation 5 provides a mathematical framework for using measured heat-to-biomass ratios to calculate product yields. In summary, biocalorimetry can valuably aid the screening and selection of (micro)organisms to be employed in bioprocesses and the optimization of their performance, as well as monitoring.

#### Screening, exploration, and selection of biocatalysts

The selection of microorganisms for biotechnological applications is guided by a range of common viewpoints that ensure their effectiveness and suitability for specific purposes. Many of the selection criteria used to evaluate microorganisms for biotechnological purposes, i.e., quantitative and/or qualitative microbial characteristics that reveal the activity or product of interest (Iris et al. 2020; Olicon-Hernandez et al. 2022), can be targeted through the use of biocalorimetry. Such selection criteria can be divided into two different types. Primary screening frequently involves rapid tests aiming at, e.g., hydrolysis or inhibition halos, pH indicator changes, or growth behavior on solid media in combination with high amounts of samples to be processed. Typically, the presence or absence of a desired activity is evaluated qualitatively (Olicon-Hernandez et al. 2022). Subsequent secondary screenings then typically apply more specific and sensitive analyses, using direct and quantitative methods and less strains to identify the most efficient candidates (Olicon-Hernandez et al. 2022). Since biocalorimetry enables the sensitive quantification of microbial growth and product formation, it is well suited to provide quantitative data when applied within secondary screenings (Braissant et al. 2013; Duong et al. 2022a, b).

Typical characteristics of microorganisms quantitatively addressed in biotechnological screening approaches involve their growth rates, nutritional requirements, e.g., with respect to carbon and nitrogen sources, and their environmental tolerance, e.g., towards temperature, pH, osmolarity, and oxygen. Further important characteristics deserving quantification relate to the absence of toxic or inhibitory by-products and the formation of desired products, such as enzymes, antibiotics, organic acids, vitamins, amino acids, steroids, siderophores, bioinsecticides, biofuels, and biomaterials like polyhydroxyalkanoates (PHAs) or polysaccharides (Olicon-Hernandez et al. 2022; Steele and Stowers 1991; Yu et al. 2019). The related necessary phenotyping of wild-type organisms—including those from extreme or so far under-explored environments—as well as engineered platform organisms and microbial cell factories (Beloqui et al. 2008; Gallo and Aulitto 2024; Hemmerich et al. 2018; Leavell et al. 2020; Olicon-Hernandez et al. 2022) opens up a very wide field of potential biocalorimetry applications aiming to quantify organismic activities or responses with respect to the parameter(s) of interest, hereby enabling to select the best suited organism(s) for a desired process.

#### Bioprocess optimization

Process optimization is a further field of potential biocalorimetry applications. For instance, to identify optimal conditions with respect to pH, temperature or substrate availability for targeted product formation can be aided by predictive modeling involving yield coefficients (Adadi et al. 2012; Rabbers et al. 2022; Wortel et al. 2018). Trade-offs frequently characterize the relationship between growth rate and biomass as well as product yield. Primary metabolites, such as amino acids, nucleotides, or organic acids, are produced during the growth phase of microorganisms. Thus, under certain conditions corresponding metabolite yields can be improved along with increased cell dry weight and growth rate upon increasing the substrate concentration (Elsayed et al. 2019). Nevertheless, maximizing the growth rate may also result in lower primary product yields whereas optimizing for product yield, e.g., through making use of overflow metabolism may slow down growth. Such effects suggest to fine-tune substrate concentrations for a desired outcome, based on an understanding of related trade-offs with the help of yield coefficients (Rabbers et al. 2022; Wortel et al. 2018).

Metabolic heat yield coefficients such as the *Y*_Q/X_ parameter introduced above do also closely reflect rate-yield trade-offs, where faster growth rates are often realized at the expense of lower biomass yields (Maskow and Babel 1998). As metabolic rates increase, energy dissipation in the form of heat can also increase and potentially lead to a higher heat yield coefficient together with lower biomass yields (Lipson 2015; Roden and Jin 2011). This trade-off highlights a balance between rapid growth and efficient substrate utilization, which microorganisms have to achieve. Moreover, different from growth-coupled product formation the onset of the formation of desired secondary metabolites usually goes along with a transition from the exponential to the stationary growth phase of a producing strain. The corresponding resource reallocation results in an increase in secondary metabolite production at the expense of cell growth, where biomass levels can be maintained at a relatively stable level by applying appropriate substrate feeding regimes under certain conditions (Baptista-Neto et al. 2000; Elsayed et al. 2019). This reallocation is reflected by shifts in the heat production rate, which can be used to control the feeding rate of the carbon substrate or essential nutrients such as the nitrogen source to a bioreactor, in order to maintain the maximum possible product formation rate without harming the biocatalyst (Rohde et al. 2016).

As for primary metabolites, yield coefficients can serve as valuable indicators for predicting optimal conditions also for secondary metabolite production. While predictive modeling approaches aiming at bioprocess optimization have been based on mass balances involving substrate, product, and biomass amounts (Eq. 3) in the past, metabolic heat-based energy balances (Eq. 5) may increasingly aid predictive modeling in the future. Successful predictive models for *Y*_X/S_ based on Gibbs energy have already been described (Liu et al. 2007; McCarty and Bae 2011; Smeaton and Van Cappellen 2018; Tijhuis et al. 1993; Trapp et al. 2022; Xiao and VanBriesen 2008).

#### Monitoring of biological processes

Biocalorimetry has emerged as a versatile analytical technique utilized across various fields (Table 1), particularly in medical and environmental microbiology research while technical-scale bioprocess monitoring applications still remain at their infancy. Early speculation already existed regarding the use of calorimetry for process monitoring as the ratio of heat-producing volume to heat-exchanging surface increases with each scale-up, thereby improving measurement accuracy (von Stockar and Marison 1991; see also Eq. 2). Building on this pioneering work, calorimetric methods have been successfully developed to monitor bioreactors of the 50 L (Regestein et al. 2013) and even of the 100 m^3^ scale (Türker 2004). Such methods were also applied for process control, e.g., in systems involving toxic methanol as a substrate (Rohde et al. 2016). Metabolic heat measurements have been proven to be instrumental for the detection and analysis of microorganisms, evaluation of microbial processes, and assessing the efficacy of antimicrobial agents (Braissant et al. 2010b). Biocalorimetry allows for the quantification of microbial activity over extended periods ranging from hours to days, hereby providing valuable insights into microbial dynamics (Braissant et al. 2010b). It further has been employed in food spoilage assessment, the evaluation of additive effects in cosmetics, investigations into the decomposition processes of organic matter in soils, and studying seed germination (Wadsö 2002). Metabolic heat measurements are also well-suited for investigating small eukaryotic organisms such as nematodes, enabling researchers to gain insights into their metabolic processes (Braeckman et al. 2002).

With respect to microorganisms, biocalorimetry has been effectively used to monitor bacterial metabolic activities, identify unexpected metabolic events (Maskow and Kleinsteuber 2004), and track metabolic shifts (Duboc et al. 1998; Maskow and Babel 1998). It enables to follow the conversion of toxic substrates into valuable products, such as biopolyesters (Maskow et al. 2006) or ectoine (Maskow and Kleinsteuber 2004), and has also been applied in controlling the feed to fed-batch fermentation systems (Rohde et al. 2016). Biocalorimetry has further been used to study fungal submerged fermentations, where it aided the elucidation of relationships between metabolic heat production rates and fungal growth kinetics, product formation, and exoenzyme secretion (Dhandapani et al. 2012; Santharam et al. 2017; Schuler et al. 2012).

While successful biocalorimetry applications could be demonstrated for numerous liquid-phase fermentation studies, research into the applicability of this analytical technique to solid-state fermentation (SSF) and the potential benefits it may offer in this context is still limited. Nevertheless, isothermal microcalorimetry (IMC) has shown promise, e.g., in the successful testing and validation of an SSF process using the *β*-carotene-producing yeast *Blakeslea trispora* during growing on barley (Altwasser et al. 2017). Here, this method effectively detected changes in growth and product formation as evidenced by the generated heat signals (Altwasser et al. 2017). Additionally, Wadsö et al. (2017b) demonstrated the successful monitoring of fungal colonization of wood materials using IMC. This approach provided more comprehensive insights into the degradation processes compared to traditional decay assessment methods, suggesting that IMC could be beneficial for both applied and fundamental studies of decay fungi. Similarly, we have successfully employed biocalorimetry during fungal SSF of lignocellulosic agricultural by-products such as wheat straw (Duong et al. 2022a, b; 2024). Despite its advantages, applying biocalorimetry to solid media comes with challenges. The complex composition of solid substrates, along with heterogeneous distributions of substances and colonization patterns, makes relationships between material inputs and energy outputs more intricate than in submerged fermentations. Such circumstances still necessitate further research into the subject of concern.

## Promising biocalorimetry applications to natural and synthetic macromolecules

### Plant biomass and organic waste valorization

The usefulness of potential biocalorimetry applications becomes particularly obvious when the increasing interest in the valorization of solid, inhomogeneous substrates in submerged or SSF-based bioprocesses comes into focus. Recent demands for a next-generation biotechnology aiming to transform current industrial biotechnology into competitive processes list—among other requests—the use of renewable low‐cost mixed substrates such as lignocellulose and other plant materials, less freshwater consumption, and the limitation or avoidance of time- and energy-demanding sterilization processes (Yu et al. 2019). Both liquid-phase (aerobic fermentation, anaerobic digestion) and SSF processes can fulfil such criteria to varying degrees, depending on the specific substrates, products, and conversion processes that are considered (Astudillo et al. 2023; Bentil et al. 2018; Bhutto et al. 2017; Okolie et al. 2021; Sulis et al. 2025). However, for both types of processes, the solid nature of substrates complicates a rapid, efficient, and reliable process data acquisition. Even for submerged processes where a high feedstock concentration is often desired (Okolie et al. 2021), sampling the water phase is only representative for sufficiently dissolved or suspended compounds.

As part of current circular bioeconomy approaches, SSF processes are considered emergent technologies for biorefineries aiming at the conversion of frequently lignocellulosic agricultural by-products and other types of organic waste currently treated in composting or anaerobic digestion processes into, e.g., green chemicals, bioplastics, textiles, adhesives, paper materials, or biofuels (Artola et al. 2024; Blánquez et al. 2022; Martínez-Avila et al. 2022; Sánchez 2024). SSF provides major advantages over other fermentation techniques such as process robustness, applicability of low-cost residual materials as substrates, reduced usage of water, and lower sensitivity with respect to sterility problems (Holker et al. 2004; Mitchell et al. 2006) but also presents significant challenges with respect to process monitoring, optimization, and control (Duong et al. 2022a; Steudler and Bley 2015). A major difficulty in SSF processes relates to the commonly inhomogeneous microbial growth on solid substrates, which hampers the rapid and efficient acquisition of reliable and representative data on microbial growth and activity (Duong et al. 2022a; Raimbault 1998). On the other hand, adequate control strategies should ideally be based on only one or a few parameter(s), enabling sufficient characterization of the overall process within reasonably short time (Raimbault 1998; Steudler and Bley 2015). In this regard, biocalorimetry appears as a valuable tool potentially replacing invasive methods for monitoring microbial growth and/or activity on solid substrates, which are based on sampling and analyses of cell components, e.g., ergosterol, glucosamine, or nucleic acids, and therefore are commonly laborious and time-consuming (Steudler and Bley 2015). Real-time monitoring of microbial growth and activity remains generally complicated if cells cannot directly be accessed or co-cultured species possess a too similar morphology, despite indirect biomass monitoring based on analyses of both soluble and volatile (bio)chemicals with the help of optical or mass spectrometry-based techniques and biosensors has been described (Dambruin et al. 2025). In this respect, biocalorimetry holds promise particularly in situations where cells are not directly accessible due to fungal peculiarities such as surface-adhering filamentous growth. Calorimetric measurements have also been suggested to aid SSF process upscaling, where the inadequate dissipation of metabolic heat is a critical factor for SSF applications at larger scale (Jian and Yang 2006; Vauris et al. 2022).

In addition to facilitating technical process control, biocalorimetry could also provide information about ecological strategies employed by saprotrophic microbial decomposer organisms during utilization of complex solid substrates. In this context, it could also be used to quantify related functional traits, i.e., characteristics that inform about the practical usability of such organisms for a given biotechnological process (Duong et al. 2022a, b).

#### Synthetic polymers in a circular economy

Man-made synthetic polymers (plastics) represent another group of solid, inhomogeneous materials with potential use as substrates in submerged fermentation or SSF-like bioprocesses, including composting (Sánchez 2024). As a part of current tremendous efforts to integrate plastic waste into a circular economy, biological methods for the breakdown of synthetic polymers to yield new useful materials and products (e.g., new polymers, further chemicals, syngas, compost, energy) are increasingly gaining attention (Bergeson et al. 2024; Kalita and Hakkarainen 2023; Nixon et al. 2024; Polo Fonseca et al. 2023). Any attempt to valorize plastic waste through its biochemical conversion would first require to decompose a solid substrate. A recent paper on this subject concludes that especially hydrolysable plastics such as polyesters, polyurethanes, and polyamides are amenable to direct enzymatic depolymerization, whereas for plastics containing only saturated carbon-carbon bonds in their backbones such as polyolefins and polystyrene, an initial (thermo)chemical polymer deconstruction followed by microbial metabolism of resulting small-sized breakdown products may pave a way to chemo-biotechnological recycling processes (Wei et al. 2025).

Enzymatic recycling of plastic waste has now become a realistic vision. It was first implemented at industrial demonstration plant scale for enzymatic depolymerization of the polyester polyethylene terephthalate (PET) contained in diverse plastic materials and textiles, allowing the recycling of PET waste and the production of completely recycled (and recyclable) PET products (CARBIOS 2025; Tournier et al. 2020). A high solid substrate loading of over 20% (w/v) is considered a prerequisite for corresponding industrial submerged enzymatic processes (Tournier et al. 2020; Wei et al. 2025). Efficient enzymatic depolymerization of high-crystallinity PET was also reported for moist-solid reaction mixtures lacking a bulk water phase (Kaabel et al. 2021), hereby resembling an SSF-like process. Biodegradable plastics potentially amenable to composting in “organic recycling” processes (Kalita and Hakkarainen 2023) are considered environmentally friendly alternatives to many commodity plastics. Nevertheless, also these materials frequently are still too inert to allow their full breakdown even at the operational conditions of thermophilic digesters (DelRe et al. 2021). Embedding polyesterase enzymes into such biodegradable plastics could successfully be demonstrated to enable their nearly complete disintegration even under home-composting conditions, hereby providing a scalable solution for the treatment of plastic-containing waste also in industrial composting and biomethane production processes (DelRe et al. 2021; Guicherd et al. 2024). It still remains to be determined whether biochemical plastic decomposition could offer a substrate specificity sufficient to enable the selective recycling of mixed plastic waste, thus overcoming the well-known related difficulties (Lai et al. 2025; Sullivan et al. 2022; Wei et al. 2025).

**Fig. 3:**
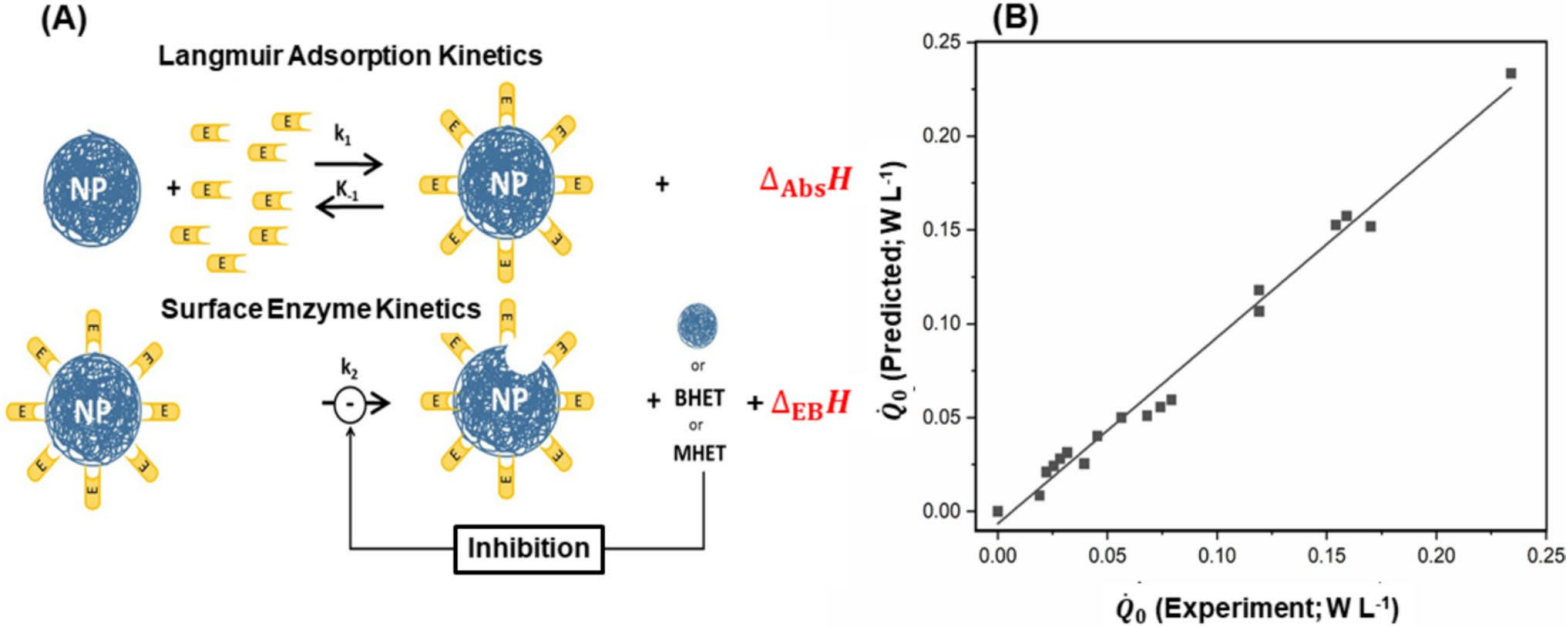
Thermokinetic model of enzymatic PET degradation and validation. **A** Kinetic-energetic scheme (NP, nanoparticle; E, enzyme) with rate constants k_1_, k_−1_, k_2_, and enthalpy terms ${\Delta}_{\mathrm{A}\mathrm{d}\mathrm{s}}H$ (adsorption) and ${\Delta}_{\mathrm{E}\mathrm{B}}H$ (ester bond cleavage). BHET (bis(hydroxyethyl) terephthalate) and MHET (mono(hydroxyethyl) terephthalate) denote soluble intermediates. In the initial step, the enzyme adsorbs to the nanoparticle surface, initiating a statistical depolymerization process. Adsorption enthalpy was obtained using inactive cutinase, while the active enzyme enabled estimation of kinetic parameters and ${\Delta}_{\mathrm{E}\mathrm{B}}H$. The model (Vogel et al. 2021) assumes rapid assumes rapid Langmuir-type adsorption followed by Michaelis–Menten kinetics. **B** Experimental (black square) vs. modeled heat production rates at time zero (${\dot{Q}}_{0}$; line), showing good agreement. Adapted with permission from Vogel et al. (2021) © 2021 The Authors. Published by Elsevier B.V.. Open access article under the CC BY-NC-ND license (http://creativecommons.org/licenses/by-nc-nd/4.0/)

Biocalorimetric process monitoring approaches so far have only rarely been applied with respect to the biological decomposition of synthetic polymers, despite the advantages of biocalorimetry for applications employing heterogeneous solid substrates such as natural macromolecules, as described above. While differential scanning calorimetry (DSC) has long been established for measuring the glass transition, crystallization, and melting of PET (Kang et al. 2011) and now is also employed to assess the quality of PET waste for recycling purposes (Sudomova et al. 2023), the application of biocalorimetry to monitor enzymatic depolymerization is a novel approach. In an initial pilot study using isothermal titration calorimetry (Vogel et al. 2021), it was shown that the heat production rate profile offers valuable insights into the reaction mechanism of PET nanoparticles degradation, the extent of depolymerization, and potential inhibitory effects. The reaction mechanism elucidated in the aforementioned study is illustrated in Fig. 3A. The comparison between modeled and experimentally determined heat production rates at the time zero (${\dot{Q}}_{0}$) demonstrates good agreement, indicating that the model performs well (Fig. 3B).

## Current challenges and limitations in terms of applicability

### Instrumental and operational limitations of IMC

Considering the most widely applied calorimetric technique, IMC, reactions are typically conducted in sealed ampoules. Under such aeration-preventing conditions, ongoing metabolic activity can result in oxygen depletion and/or CO₂ accumulation. Such changes may alter the use of terminal electron acceptors, induce fermentative metabolic pathways, inhibit microbial growth, or even create toxic conditions. As a consequence, the recorded thermal signal may become distorted and no longer accurately represent the actual biological process under investigation.

These limitations can, in principle, be mitigated by periodically opening the reaction vessel to restore gas exchange. The oxycaloric equivalent (between −430 and −480 kJ mol^−1^; Hansen et al. 2004) enables the estimation of oxygen consumption from the measured heat output, which can then be compared with the oxygen initially available in the ampoule, calculated using the ideal gas law. This approach allows the determination of appropriate intervals for venting or reopening the vessel. However, removing ampoules from the calorimeter introduces thermal disturbances that may persist from several minutes to hours, depending on sample size and instrument configuration. During this recovery period, reliable heat flow measurements cannot be obtained.

An alternative strategy involves the use of a continuous gas flow of water vapor-saturated air to maintain stable aerobic conditions. Usually, implementing such an approach requires the modification of commercially available ampoules. Moreover, the signal quality may be reduced due to additional thermal bridges introduced by tubing connections and potential condensation effects within the gas-transfer system. In principle, semi-permeable membrane caps can provide another solution by enabling selective exchange of O₂ and CO₂ while minimizing water loss from the system.

#### Mixed culture scenarios

The simultaneous activity of two or more populations of (micro)organisms together contributing to a desired bioprocess may pose particular challenges to process monitoring and calorimetric data interpretations. Corresponding mixed or co-cultures (for simplicity we here use all terms of this context interchangeably but also are aware of current requests for a common nomenclature; Sanita Lima and Coutinho de Lucas 2021) may be composed of known species or may partly or even fully contain or be dominated by unknown species. Populations of different species of a mixed culture further can represent just one organismic kingdom, e.g., Bacteria, or may belong to different kingdoms, such as Bacteria, Archaea, Fungi, or Protozoa (kingdom classification according to Ruggiero et al. (2015)). The view that co-cultivations held promise for a wide range of applications in biotechnology and medicine and also can support ecological research is now widely accepted (Kapoore et al. 2022). Co-cultures are employed, e.g., in screenings for microbial secondary metabolites with anti-microbial activity (Tironi et al. 2024) or further therapeutic value (Selegato and Castro-Gamboa 2023) or to aid the development of biorefinery platforms (Rosero-Chasoy et al. 2021). Considering lignocellulose-based biorefineries, recent research indicates that compared to microbial monocultures, the use of microbial co-cultures can enhance the delignification of lignocellulosic materials and hereby improve the in vitro fermentation index, which includes factors such as digestibility, volatile fatty acids, and gas production (Datsomor et al. 2022). Constructed fungal consortia have repeatedly been demonstrated to improve delignification and enhance digestibility when applied for pretreatment of lignocellulosic agricultural by-products and hereby outperform the respective individual fungal strains they were built upon (Chen et al. 2019; Hermosilla et al. 2018; Mewada et al. 2017).

Beyond co-culturing exclusively known species, e.g., in the form of constructed microbial consortia, many bioprocesses are operated under non-sterile conditions. Such non-sterile bioprocesses rely on conditions that favor the activity of one or more desired (micro)organism(s), which may be known and sometimes are added from outside, over potential contaminants without completely eliminating the latter. Wastewater treatment (Parret et al. 2025), biological treatment of organic environmental contaminants in diverse bioremediation schemes (Omeroglu et al. 2025), the production of microbial enzymes (Pan et al. 2019), and the microbial conversion of renewable feedstocks into biofuels and platform chemicals are typical examples for non-sterile bioprocesses aiming at competitiveness through high efficiency along with reduced costs, robustness, reduced maintenance efforts, comparatively simple bioreactor design, and ease of operation (Chen and Wan 2017; Chitnis et al. 2024). Also, the bioprocess-based industrial-scale valorization of lignocellulosic and other solid agricultural wastes ideally needs to operate under non-sterile conditions, thus questioning pure culture approaches (Galbe and Wallberg 2019; Vasco-Correa et al. 2019; Zanellati et al. 2020).

For all of the aforementioned scenarios, the simultaneous colonization of substrates by multiple (micro)organisms or enzymes and their activity can result in complex interactions and dynamics that may vary in response to species combinations and substrate types (Hiscox et al. 2015; Song et al. 2012; Woodward and Boddy 2008). To monitor and control processes involving co-cultures or consortia via biocalorimetry is hence expected to be considerably more challenging than coping with monocultures or may even not work at all, which still needs to be figured out in the future. The aforementioned nonspecificity of calorimetric signals would certainly present a serious weakness for related approaches. Further complications related to data interpretation arise from the fact that heat signals depend on both the carbon and energy use efficiency of the individual community members, and on their respective metabolic rates. To make fundamental progress in this area, thermokinetic analyses of synthetic communities would be advisable. On the other hand, however, the elemental composition and energy contents of various microbial species are very similar (Popovic 2019), which simplifies the interpretation of heat signals. Also, different growth dynamics of the members of a microbial community may result in heat signals displaying several maxima over time, which could facilitate the interpretation of such data.

Despite the uncertainties mentioned before, calorimetric data derived from microbial co-cultures or consortia could enhance our understanding of microbial interspecies interactions, thereby providing valuable insights for microbial ecology research. Similar to bioprocesses involving only partly or not further defined mixed cultures, biocalorimetric methods aimed at studying ecological processes involving (micro)organisms still require validation within a community context (Crowther et al. 2014; Duong et al. 2022b; Lustenhouwer et al. 2020).

#### Completion and availability of metabolic heat measurement-derived functional data

In order to aid the selection of suitable strains for bioprocesses, the still limited availability of metabolic heat measurement-based data on strains’ capabilities, performances, and requirements highlights the necessity to collect and maintain more of such functional data. The biocalorimetry-aided provision of information about ecological strategies of (micro)organisms of potential biotechnological interest and the definition and quantification of related functional traits, i.e., characteristics that inform about the practical usability of such organisms for a given biotechnological process, has been suggested for saprotrophic decomposer fungi utilizing complex solid substrates (Duong et al. 2022a, b). The exploration of metabolic heat-derived parameters of organisms, e.g., species-, substrate-, and metabolism type-specific *Y*_Q/X_ values as mentioned before (Birou et al. 1987a; Duong et al. 2022a, b; Katla et al. 2020; Mohan and Sivaprakasam 2017; Schuler et al. 2012; Sivaprakasam et al. 2011), should be expanded in order to encompass a broader range of species of potential biotechnological interest. This expansion is crucial as related data is still scarce. Additionally, valuable insights can be gained from (micro)organisms inhabiting different types of ecosystems, e.g., marine, freshwater, and mangrove, as well as from organisms with different lifestyles, such as saprobionts, symbionts, pathogens, or parasites.

In the context of biotechnological applications, having access to specific parameters based on metabolic heat measurements could assist in selecting the most suitable organism(s) for a specific purpose. Corresponding metabolic heat-related parameters could be integrated into existing, often primarily ecology-motivated databases such as FungalTraits, FUNGuild and FunFun for fungi (Põlme et al. 2021; Zanne et al. 2020), or BactoTraits (Cébron et al. 2021), BacDive (Schober et al. 2025), and the bacterial and archaeal phenotypic trait database (Madin et al. 2020) in case of bacteria and archaea. Beyond biotechnological applications, integrating such data may also enhance functional assignments of microbial community members and improve the ecological interpretation of environmental studies.

## Prospect

Biocalorimetry presents a powerful, versatile, and scalable approach for advancing the biotechnological utilization of both natural and synthetic macromolecules, particularly addressing the challenges posed by complex solid-state systems on the basis of plant biomass and its residues as well as plastic waste. Its capability to offer real-time measurements of metabolic heat production enables sensitive monitoring and optimization across diverse substrates and scales ranging from microliters to cubic meters. By directly measuring energy flows, biocalorimetry overcomes limitations inherent in disruptive methods especially in heterogeneous and solid materials, where representative sampling is difficult. This direct measurement of enthalpy changes provides a reliable basis for quantifying microbial activity, depolymerization completeness, and reaction rates, which can be translated into biologically meaningful parameters such as biomass growth and product formation rates. The replacement of time-consuming and laborious invasive measurements, e.g., analyses of cell components, by calorimetry offers the possibility to accelerate bioprocess development, scale-up, and real-time quality control. Moreover, it can provide valuable ecological insights by quantifying functional traits associated with microbial resource utilization strategies and thus inform strain selection and bioprocess design in ecologically relevant and industrially applicable manners.

High-added-value bioproducts and biomaterials that can be derived from various types of biomasses and related agro-industrial waste are currently gaining increasing attention (Monteiro et al. 2023; Perea-Moreno and Muñoz-Rodríguez 2024; Vallejos et al. 2024) and therefore represent obvious potential areas of application for biocalorimetry. Likely even more challenging, the application of calorimetry to bioprocesses targeting synthetic polymers aims at a frontier with significant potential but comparatively limited exploration. Nevertheless, enzymatic depolymerization and microbial conversion for plastic waste valorization are nowadays emerging as realistic solutions for addressing the environmental and resource challenges posed by persistent plastic pollution. Biocalorimetry has begun to elucidate the kinetics and thermodynamics of enzymatic degradation of synthetic polymers and hereby can help to reveal mechanisms of action, extents of depolymerization, and potential inhibitory influences in real time. Such insights are critical for optimizing enzymatic recycling processes at industrially relevant scales and could contribute to the advancement of composting strategies for biodegradable plastics, thus potentially aiding in the development of environmentally friendly circular approaches to plastic waste treatment.

Limitations and therefore challenges remain for extending biocalorimetric techniques to mixed microbial cultures, consortium-based bioprocesses, and non-sterile operational environments—settings where microbial interactions and dynamic population changes add complexity to signal interpretation and process control. Nevertheless, the integration of metabolic heat-based data on microbial functional traits into comprehensive databases could enhance process monitoring capabilities and deepen the understanding of interspecies interactions, thereby supporting the design of more robust and efficient mixed-culture bioprocesses. Progress in this field demands further methodological development, extensive validation in complex community contexts, and the building of large, strain-specific datasets of metabolic heat parameters across a broad range of microorganisms and substrate types. Expanding such datasets will not only facilitate the selection of appropriate biocatalysts for defined biotechnological objectives but also improve ecological interpretations of microbial community functioning in natural and engineered systems.

In addition, recent studies have highlighted the advantages of combining experimental data from multiple -omics platforms. For instance, genomics, transcriptomics, proteomics, and fluxomics can provide crucial information about enzymes (Pang et al. 2022; Picard et al. 2021; Worheide et al. 2021). When such data are integrated with metabolomics, they may help to uncover the underlying causes of metabolic alterations (Go et al. 2024). Furthermore, since genomics, transcriptomics, and fluxomics encompass data on all genes/transcripts or utilize models based on these datasets, they enable comprehensive investigation of metabolic changes (Rau et al. 2022; Worheide et al. 2021). With regard to this, the future combination of biocalorimetry with -omics technologies and data-driven thermodynamic modeling holds promise for the interpretation and prediction of complex biological processes. The incorporation of machine learning and advanced computational models may enable predictive capabilities that can optimize bioprocess monitoring and control, thus facilitating the development of automated and sustainable bioprocesses with improved resource efficiency and productivity. This multidisciplinary approach could potentially accelerate innovation in bioprocess engineering by delivering scalable and environmentally conscious solutions.

Biocalorimetry is well positioned to accelerate research and industrial innovation. It offers a technology platform for optimizing resource utilization, enhancing process robustness, and fostering circular approaches to bio-based production and waste remediation. Metabolic heat measurements may also become applicable to miniaturized high-throughput approaches for testing, improving, or monitoring the performance of potential production strains in accelerated bioprocess developments (Hemmerich et al. 2018; Leavell et al. 2020; Wehrs et al. 2020). Multi-parallel biocalorimeters equipped with up to 48 channels for data recording are already available today (Wadsö et al. 2017a), which may be accordingly adapted to related high-throughput screening platforms in the future.

In sum, biocalorimetry holds reasonable promise for enabling the next generation of sustainable biotechnologies aligned with environmental stewardship and the efficient harnessing of biological resources. Though, realizing its full potential will hinge on overcoming current limitations relating to complex substrate matrices, mixed microbial communities, and data availability. Continued interdisciplinary efforts bridging thermodynamics, microbiology, ecology, and engineering will be vital for advancing this field and integrating biocalorimetry into mainstream industrial and research applications.

## Data Availability

No new data were generated in this study. All information analyzed and discussed is available within the referenced sources.
